# Hospital staff perspectives towards health technology assessment: data from a multidisciplinary survey

**DOI:** 10.1186/s12961-019-0469-3

**Published:** 2019-07-23

**Authors:** Orna Tal, Meirav Booch, Sara Bar-Yehuda

**Affiliations:** 1 Shamir Medical Center (Assaf Harofeh), 70300 Zerifin, Israel; 2 ICET - Israeli Center for Emerging Technologies, Zerifin, Israel; 3 BioMedWrite, Zerifin, Israel

**Keywords:** Hospital-based health technology assessment, multi criteria decision analysis, hospital personnel survey

## Abstract

**Background:**

Technology adoption in hospitals is usually based on cost-effectiveness analysis, feasibility and potential success. Different countries have embraced a range of principles to accomplish an effective comprehensive process of health technology assessment (HTA). The aim of the study was to analyse the viewpoints and relative weight of technology-oriented hospital staff members toward the clinical, social, technological and economic aspects of HTA.

**Methods:**

Using a structured questionnaire, a survey was conducted among different professionals in an 850-bed hospital.

**Results:**

We revealed a range of viewpoints among hospital staff members according to their personal characteristics and professional standpoints. The clinical aspects of HTA were considered ‘highly important’ (HI) by most participants, especially the ‘lifesaving’ parameter. Similarly, the ‘lack of effective alternative technology’ was ranked HI by a high percentage of participants, independent of their profession. Economic aspects were ranked HI only by half of the participants, while social and technological aspects were ranked HI only by a relatively low percentage. Nurses added ‘improving quality of life’, ‘increasing teamwork efficiency’ and ‘improving medical standards’. Allied health professionals focused on ‘lack of effective alternative technologies’ as a main argument for adoption of HTA, alongside increasing efficiency, budget savings and contribution to hospital reputation. Engineers emphasised the requirement of significant investment in infrastructure and increasing efficiency. Administrators ranked patient experience as HI. Interestingly, the high ranking of social aspects correlated with older responders, while junior staff ranked safety significantly higher.

**Conclusions:**

A multi-perspective multidisciplinary approach would be beneficial for policy-makers at hospitals and even on a national scale in Israel.

## Introduction

Health technology assessment (HTA) is a scientific field designed to establish tools for the acquisition, use or exchange of medical technologies [1]. The Office of Health Technology Assessment defined the term ‘medical technology’ as “*drugs, devices, and diagnostic and therapeutic approaches (general and surgical procedures), as well as administrative frameworks that provide health care*” [2, 3]. The methodology of HTA spread around the world in the late 1980s [4–6].

The International Network of Agencies for Health Technology Assessment and Health Technology Assessment International deal with strategic issues concerning the present status of HTA, its development and implications for healthcare systems, industry, patients and other stakeholders [3, 7].

The objective in performing HTA is to provide information about treatment alternatives to policy- and decision-makers (Ministry of Health and Government), to medical organisations that provide healthcare services (hospitals or health maintenance organisations), to insurers responsible for the funding, to healthcare practitioners (physicians, nurses, etc.), and to patients and their families [1, 8].

HTA is an inclusive, multidisciplinary scientific process, yielding a profitability estimation of the medical and economic outcomes to the patient and society upon adoption of a specific technology [9]. This estimation takes into consideration various medical and economic variables, such as prevalence of the disease, relevant target population, cost effectiveness and value added, costs, as well as the health system burden of each new technology [10].

Traditionally, an effort has been made to base decisions to adopt hospital technologies on evidence-based medicine, assuming that medical executives translate their knowledge into daily decision-making. In the case of innovations, as uncertainty is dominant, the rule of work suggests that managers lean on the right HTA principles and consult clinicians to support their decisions using the current best evidence. Ensuring decisions are consistent with patient values and preferences is even more challenging, and guidelines to approach this issue have not yet been achieved [11].

In order to assess the comparative benefits of several technologies used for the same purpose, and choose the most advantageous and efficient option, a set of common standard definitions has to be formed [1]. Indeed, in the early 1990s an expanding estimation model was accepted in most countries [12, 13], as well as in Israel. This model included laboratory tests, nanotechnology and disposable equipment. Since 2000, the model also includes information, data and communication systems.

Over the last decade, the need to assess the efficiency of a health technology has been strengthened, especially in hospitalisation institutions, due to their limited resources and the continuous stream of innovative promising technologies arising on a daily basis. The importance of specific HTA in hospitals arises from the unique characteristics of the hospital environment – hospitals are the ‘port of entry’ of new technologies in the healthcare system. Executives face the challenge of effectively adopting innovations with the need to improve rationality of decision-making, the urge to do so in a limited timeframe and with limited resources, while assuring an appropriate risk-benefit balance. The concept of an evaluation mechanism fosters a culture of targeted assessment that integrates scientific and local evidence, combined with practices and management decisions [14].

Therefore, in 2006, hospital-based HTA (HB HTA) emerged to promote HTA at the hospital level, initiating a unique methodology based on integration of the following principles: providing focused information for hospital decision-makers, aiming to define leadership and partnerships, meeting with strategy of HB-HTA units and targeting the economic aspect to allocate adequate resources that ensure the operation of HB-HTA units. Monitoring, through measurement of the short- and long-term impact of the overall performance of HB-HTA units, is important feedback for the mechanisms relating to the expertise of the HB-HTA unit [15].

Nevertheless, HB-HTA varies from one hospital to another and from country to country. The worldwide experience is that the model of HB-HTA is also influenced by the level of integration with other HTA bodies at the national, regional or provincial levels, shaping the HB-HTA approach [16]. Four major approaches appeared, including an integrated specialised HTA unit, a stand-alone HTA unit, an integrated-essential HTA and an independent group unit. Moreover, efforts have been made to spread and share knowledge regarding the use of HTA in the unique circumstances of hospitals, for example, the activity conducted by AdHopHTA (a European project on hospital-based health technology assessment) [17].

Rosenstein et al. [18] found that 90% out of 19 hospitals in the western part of the United States reported the existence of an organised committee responsible for the evaluation process of new technologies, mostly by a proactive HTA process. In many hospitals, the process is neither standardised nor regulated, but rather forms part of a 3- to 5-year general development plan of the institution. Though theoretical guidelines for the process are published, the committee composition is flexible and various representatives may be included – health professionals (physicians, nurses, physiotherapists, dieticians and other caregivers), engineers, bio-technologists, economists, epidemiologists, information specialists, law and ethics professionals, and representatives of patients and the public [18]. Additionally, it is important to note that the majority of HTA is still executed at a national, regional or even local level.

In Israel, a structured national HTA mechanism for established health technologies has existed for almost two decades, mostly in the community setting [7]. The decision-making mechanism is based on defined criteria. An early alert system for emerging technologies operates on a national level by an assessment recommendation body for governmental authorities [19].

In the hospital setting, a group of 11 hospitals, supervised by the governmental division of medical centres with equal influence, simultaneously adopted similar doctrines. Although the basic HTA principles are maintained, HTA in these hospitals is still performed separately without a unified methodological framework, while agility plays a role in the dynamic world of medicine. For example, new approaches enlighten the incorporation of the volume and characteristics of patients, and economic considerations may vary when a new technology is adopted under unique circumstances.

Since the beginning of the 2000s, an HTA committee has been operating at Shamir Medical Center, integrating essential HTA rules alongside principles of clinical benefit, cost and feasibility, presenting its recommendations to the hospital director, who decides about new technology acquisition. The HTA committee includes 16 members – five physicians, two medical directors (one of them a technology assessor), two senior clinical experts (in gastro and ENT), one infectious control expert, three nurses (surgery, risk management, reuse and sterilisation expert), two medical occupation experts, two bio-technicians/engineers, two economists, and two purchasing representatives. The committee meets quarterly on a regular basis to assess new technologies that are candidates for implementation by the hospital management. The members discuss 10–12 new technologies at each meeting; mostly medical devices (72%), procedures (up to 15%), and surgical equipment and laboratory tests. Of note, new drugs are discussed via a different mechanism, as in Israel they are supplied by the Health Maintenance Organizations (the public insurer). The new technology is presented by the physician that requires the utility and/or has experience in using it. A short assessment is conducted prior to the committee meeting by the HB-HTA unit (a technology assessor physician, a quality assurance physician/nurse, and an economist with the support of an informational specialist). Their summary, containing published evidence as well as real world experience, is presented to the committee focusing on the advantages, challenges, barriers and feasibility for local adoption [20]. The technology is compared to alternatives and costs are estimated (including infrastructure requirements, maintenance costs, relevant population needs and insurance coverage, all of which reflect demand and affordability). The recommendations are presented to the hospital executive management for approval and budget allocation. Prioritisation is based on medical effectiveness and skilled experts that inspire clinical excellence and resources. The average annual adoption rate is 55% (~24/44) [21].

The mounting costs of new technologies and their handling demands, the need for skilled and experienced personnel, as well as budget constraints, have led to the necessity for a more complex HTA process.

Thus, the aim of the present study was to examine the positions of various hospital staff members regarding the HTA process performed in a public governmental medical centre, and to analyse the weight of its components in relation to the adoption of new technologies.

## Methods

A survey was conducted at Shamir Medical Center (Assaf Harofeh), a general, public 850-bed hospital.

### Study design

A prospective survey was conducted during a 3-month period (July–September 2013). Four or five reminders were sent to the selected candidates to participate in the study.

### Recruitment and selection of participants

The recruitment and selection of participants was based on individuals who were involved in the committee’s activity, namely physicians, nurses, engineers and administrative personnel. The ‘administrative group’ included workers that were involved in purchasing or installing medical devices.

The study population was chosen out of the entire hospital workers group (~3500 workers, including 821 physicians, 1244 nurses and 1435 non-clinical staff at that time).

Inclusion criteria were participants that were familiar with the HTA process, as part of assessing medical technologies prior to acquisition, and were involved in the activity of HTA and technology adoption in the year prior to the survey (June 2012–May 2013).

The exclusion criterion was an inability to complete the questionnaire due to language or comprehension barriers (2 participants).

Altogether, at the beginning, we targeted 338 hospital professionals (204 senior physicians, 85 nurses (supervisors and head nurses), 8 allied professionals, 10 engineers and 31 administrators were potential participants) and 71 responded, following three reminders.

A pre-list of participants was prepared in order to selectively choose the suitable participants.

The survey questionnaire was disseminated by internet via mailing lists (based on mailing lists used by the HB-HTA unit) accompanied by an explanatory letter, which included the wide definition of ‘technology’ [22], to five groups of participants: physicians, nurses, engineers, administrative staff and allied professionals (social workers, physiotherapists, dieticians).

### Data collection

Data was organised using a Google-docs database, forming an Excel sheet.

#### The questionnaire

The questionnaire consisted of 54 questions, including five demographic details, with Hebrew being the formal language used. The questionnaire referred to the ranking of considerations towards the adoption of new technologies in a hospital setting. The collection of considerations that were used in order to create this mini-HTA tool was based on those used by Sampietro-Colom et al. [23] , who also aimed to develop a decision support tool for HTA. In the first section, the participants were asked to rank these considerations according to their relative ‘weight’, from ‘highly important’ (HI) (ranked 10) to ‘less important’ (ranked 1).

These considerations were divided into the following aspects: clinical aspects, which included lifesaving, quality of life improvement, patients’ functional improvement and safety (10 items); social aspects, including treating a large population, treating a specific population within the hospital, improving patient experience, and aiming at patient preference (11 items); technological aspects, including lack of effective alternative technology, supplementary technology to a technology already existing at the hospital, enhancing innovation and improving medical standards, and improving hospital reputation (5 items); and economic aspects, including increasing efficiency, person-power savings, budget savings, requirements for a significant investment in infrastructure, maintenance costs, and need for extensive education (10 items). Demographic characteristics (age, gender, profession and seniority) were collected for each participant (5 items). Thirteen items referred to the committee structure, assembly and mechanism of action.

A pre-test was conducted among 25 physicians to validate the Hebrew version.

### Statistical analysis

Statistical analysis was performed by SAS (version 9.4). If the frequency for a question was more than 6 we used the χ^2^ test for comparisons. If the frequency for a question was less than 6 we used the fisher test for comparisons. *P* values of less than 0.05 were defined as statistically significant.

## Results

A total of 71 participants answered the questionnaire (21% compliance), including 29 physicians, 20 nurses, 12 administrative staff, 6 engineers, and 4 allied professionals. Analysing the ranking of the criteria by the survey’s participants according to importance, the percentage of responses ranked HI (9–10) was evaluated by the different aspects and criteria.

### Evaluation by aspects of benefit (Table 1)

Overall, the importance of clinical assessment was ranked as HI by 88% of participants, whereas the importance of the economic, technological and social aspects were ranked as HI by 67%, 32% and 25%, respectively.

**Table 1:** Evaluation by aspects of benefit

Aspect of benefit	Criteria	“Highly important” ranking (Percent)
Clinical	Life saving	77
	Quality of life improvement	51
	Patients' functional improvement	30
	Safety	54
Social	Serving a large population	20
	Serving a targeted population within the hospital	13
	Improving patient experience	51
	Patients' preference	14
Technological	Lack of effective alternative technology	70
	Complemetation of a technology that already exists at the hospital	23
	Innovation and Improving medical standards	33
	Improving hospital reputation	30
Economical	Efficiency	51
	Manpower savings	43
	Budget savings	32
	Extent education	20
	Technology that requires a significant investment in infrastructure	20
	Maintenance costs	10

Among the clinical criteria, technologies that were graded HI by a high percentage of the participants were ‘lifesaving’, ‘safety’ and ‘improving quality of life’ (77%, 54% and 51%, respectively), among the social criteria, ‘improving patient experience’ was raked as HI by 51% of participants, among the technological criteria ‘lack of alternatives’ was ranked as HI by 70%, and among the economic criteria, ‘efficiency’ and ‘person-power savings’ were ranked as HI by 51% and 43%, respectively.

### Distribution of the criteria ranking by profession (Tables 2 and 3)

Physicians ranked as HI mostly clinical aspects (‘lifesaving’ 93%, ‘safety’ 74%, and ‘quality of life improvement’ 56%). Technological aspects, namely ‘lack of effective alternative technology’, was ranked as HI by 74% of physicians, whereas 33% and 37%, respectively, ranked social and economic aspects as HI.

**Table 2:** Distribution of the criteria ranking by profession

Apect of benefit	Criteria	Profession					*P* value
		"Highly important" ranking %(n)					
		Physician	Nurse	Allied health	Engineers	Administrators	
Clinical	Lifesaving	93(27)	74(15)	75(3)	67(4)	58(7)	0.08
	Quality of life improvement	56(16)	58(12)	25(1)	33(2)	50(6)	0.63
	Patients' functional improvement	26(7)	48(10)	50(2)	0(0)	25(3)	0.24
	Safety	74(20)	42(8)	25(1)	33(2)	42(5)	0.09
Social	Serving a large population	22(6)	26(5)	25(1)	0(0)	8(1)	0.22
	Serving a specific population within the hospital	11(3)	11(2)	25(1)	17(1)	17(2)	0.96
	Improving patient experience	33	74(15)	50(2)	33(2)	67(8)	0.07
	Patients' preference	11(10)	16(3)	50(2)	0(0)	17(2)	0.35
Technological	Lack of effective alternative technology	74(20)	68(14)	100(4)	67(4)	58(7)	0.41
	Completion of a technology already exists at the hospital	7(2)	42(8)	0(0)	67(4)	16(2)	0.0076
	Innovation and Improving medical standards	19(6)	58(12)	25(1)	17(1)	42(5)	0.08
	Improving hospital reputation	15(4)	32(6)	25(1)	67(4)	50(6)	0.09
Economical	Efficiency	37(11)	58(12)	25(1)	83(5)	67(8)	0.14
	Manpower savings	37(11)	58(12)	25(1)	33(2)	50(6)	0.57
	Budget savings	19(6)	26(5)	25(1)	67(4)	50(6)	0.12
	Technology that requires a significant investment in infrastructure	15(15)	5(1)	50(2)	100(6)	17(2)	<0.001
	Maintenance costs	7(2)	5(1)	25(1)	17(1)	17(2)	0.76
	Extent education	11(10)	32(6)	25(1)	33(2)	17(2)	0.56

**Table 3:** Criteria which were ranked HI by the highest percentage by each profession type

Profession	Apect of benefit (%/n)			
	Clinical	Social	Technological	Economical
Total	Lifesaving (77/55)	Improving patient experience (51/36)	Lack of effective alternative technology (70/50)	Efficiency (51/36)
Physician	Lifesaving (93/27)	Improving patient experience (33/10)	Lack of effective alternative technology (74/22)	Efficiency + Manpower savings (37/11)
Nurse	Lifesaving (74/15)	Improving patient experience (74/15)	Lack of effective alternative technology (68/14)	Efficiency + Manpower savings (58/12)
Allied health	Lifesaving (75/3)	Improving patient experience (50/2)	Lack of effective alternative technology (100/4)	Budget savings +Technology that requires a significant investment in infrastructure (50/2)
Engineers	Lifesaving (67/4)	Improving patient experience (33/2)	Lack of effective alternative technology + Completion of a technology already exists at the hospital (67/4)	Technology that requires a significant investment in infrastructure (100/6)
Administrators	Lifesaving (58/7)	Improving patient experience (67/8)	Lack of effective alternative technology (58/7)	Efficiency (67/8)

Most of the nurses ranked ‘lifesaving’, ‘improving patient experience’ and ‘lack of effective alternative technologies’ as HI (74%, 74% and 68%, respectively). A lower percentage of the nurses ranked as HI the paratemeters of ‘improving quality of life’, ‘patient functional improvement’, ‘innovation and improving medical standards’, ‘efficiency and work time reduction of the clinical staff’, and ‘manpower savings’ (58%, 48%, 58%, 58%, and 58% respectively).

Most of the allied health professionals ranked ‘lifesaving’ and ‘lack of effective alternative technologies’ as HI (75% and 100%, respectively). The economic and social criteria were all ranked as HI by 50% or less.

Engineers ranked the ‘requirements for significant investment in infrastructure for technology adoption’, ‘efficiency’, ‘lifesaving’, ‘lack of effective alternative technologies’, ‘complementary technology’, ‘budget savings’ and ‘contribution to hospital reputation’ as HI (100% (*P* <0.001), 83%, 67%, 67%, 67%, 67% and 67%, respectively). The majority of the engineers did not rank any social aspect as HI.

Most of the administrators ranked ‘improving patient experience’ and ‘efficiency’ as HI (67% and 67%, respectively). ‘Lifesaving’ and ‘lack of effective alternative technology’ were both ranked as HI by 58% of the administrators.

Table 3 summarizes and presents the criteria that were ranked as HI by the highest percentage by each profession type.

### The impact of demographic and personal characteristics

#### Age (Table 4)

Comparing ranking by two age groups (20–39 and 40+ years) revealed that the social and economic aspects were ranked as HI by more participants aged over 40 years in comparison to the younger group. There were no significant differences in the ranking of clinical and technological criteria.

**Table 4:** Evaluation of aspects and criteria by age

Apect of benefit	Criteria	Age		*P* value
		20-39	40+	
Clinical	Lifesaving	74	78	0.69
	Quality of life improvement	52	50	0.86
	Patients' functional improvement	22	35	0.27
	Safety	57	52	0.73
Social	Serving a large population	13	24	0.29
	Serving a specific population within the hospital	9	15	0.45
	Improving the serves to the patient	39	57	0.17
	Patients' preference	9	17	0.33
Technological	Lack of effective alternative technology	70	70	1
	Completion of a technology already exists at the hospital	27	22	0.69
	Innovation and Improving medical standards	30	35	0.72
	Improving hospital reputation	26	33	0.58
Economical	Efficiency	49	52	0.73
	Manpower savings	22	54	0.01
	Technology that requires a significant investment in infrastructure	33	67	1
	Maintenance costs	33	67	0.78
	Extented education	33	67	0.02

The criteria ‘person-power savings’ and ‘extended education’ were statistically significantly ranked as HI by the older group (54% in the 40+ years group vs. 22% in the 20–39 years group (*P* = 0.01) and 67% in the 40+ years group vs. 33% in the 20–39 years group (*P* = 0.02)).

#### Occupation (Table 5)

Among the participants, five declared their main daily occupation was research and were defined as ‘researchers’. Analysing the participants’ responses by their main occupational orientation revealed that, among the technological factors, ‘lack of effective alternative technologies’ was ranked as HI by a much higher percentage of clinicians in comparison to administrators and physicians who declared that their main occupation was research targeted (‘researchers’) (96%, 50% and 64%, respectively; *P* = 0.0007). ‘Patient experience’ and ‘patient preference’ were ranked as HI by a higher percentage of the research-targeted group, compared to clinicians. There were no significant differences in ranking of clinical and social criteria. The economic aspects were ranked as HI by a higher percentage of administrators, especially the criterion ‘budget savings’ (24% of the clinicians, 47% of the administrators and 10% of the researchers; *P* = 0.03). The factor ‘requires significant investment in infrastructure for technology adoption’ was also ranked as HI by a significantly higher percentage of administrators (34% of the administrators, 27% of the researchers and 4% of the clinicians; *P* = 0.02).

**Table 5:** Evaluation of aspects and criteria by occupation

Apect of benefit	Criteria	Occupation Section			*P* value
		"Highly important" ranking by Percent			
		Clinical	Administration	Research targeted	
Clinical	Lifesaving	81	75	73	0.82
	Quality of life improvement	62	47	37	0.32
	Patients' functional improvement	31	31	28	0.97
	Safety	38	46	16	0.23
Social	Serving a large population	19	19	27	0.82
	Serving a specific population within the hospital	8	13	28	0.28
	Improving the serves to the patient	54	60	18	0.06
	Patients' preference	16	19	0	0.31
Technological	Lack of effective alternative technology	96	50	64	0.0007
	Completion of a technology already exists at the hospital	19	28	18	0.66
	Innovation and Improving medical standards	31	41	18	0.37
	Improving hospital reputation	19	41	27	0.21
Economical	Efficiency	54	57	28	0.23
	Manpower savings	54	41	28	
	Technology that requires a significant investment in infrastructure	4	34	27	0.02
	Maintenance costs	4	19	0	0.08
	Extent education	15	28	10	0.29

#### Gender (Table 6)

The survey included 37 male and 34 female participants. Among females, a higher percentage of HI ranking was noted for the clinical aspects, significantly the criterion ‘patient functional improvement’ (42% of females vs. to 19% of males; *P* = 0.04). For the social aspect, a higher percentage of females ranked all the criteria as HI, highlighting statistically significant differences, especially for two criteria, namely ‘treating targeted populations’ (24% of female vs. 3% of male; *P* = 0.008) and ‘improving patient experience’ (64% and 39%, respectively; *P* = 0.04). For the technological aspect, a significantly higher percentage of females ranked the criteria of ‘enhancing innovation and improving medical standards’ as HI (48% female vs. 19% male; *P* = 0.01). No difference between genders was noted in the ranking of economic aspects.

**Table 6:** Evaluation of aspects and criteria by gender

Apect of benefit	Criteria	Gender		*P* value
		"Highly important" ranking by Percent		
		Male	Female	
Clinical	Lifesaving	78	76	0.84
	Quality of life improvement	44	58	0.28
	Patients' functional improvement	19	42	0.04
	Safety	56	51	0.74
Social	Serving a large population	14	27	0.17
	Serving a specific population within the hospital	3	24	0.008
	Improving the serves to the patient	39	64	0.04
	Patients' preference	11	18	0.4
Technological	Lack of effective alternative technology	75	64	0.31
	Completion of a technology already exists at the hospital	22	24	0.84
	Innovation and Improving medical standards	19	48	0.01
	Improving hospital reputation	31	30	0.98
Economical	Efficiency and work time reduction of the medical staff	50	52	0.9
	Manpower savings	42	45	0.75
	Budget savings	28	36	0.44
	Technology that requires a significant investment in infrastructure	28	15	0.2
	Maintenance costs	8	12	0.6
	Extent education	14	27	0.17

#### Seniority (Table 7)

‘Safety’ was ranked as HI by 82% of workers with 1–5 years of experience versus by 39% among those with 6–15 years of experience and 50% among those with 16+ years of experience (*P* = 0.02). The factor ‘person-power savings’ was statistically significantly ranked as HI by a higher percentage of those with 16+ years of experience in comparison to those with 1–5 or 6–15 years of experience (50% and 17%, respectively; *P* = 0.02).

**Table 7:** Evaluation of aspects and criteria seniority

Aspect of benefit	Criteria	Seniority (years)			*P* value
		"Highly important" ranking by Percent			
		1.-.5	6.-.15	16+	
Clinical	Lifesaving	84	69	77	0.35
	Quality of life improvement	59	42	54	0.53
	Patients' functional improvement	24	19	46	0.08
	Safety	82	39	50	0.02
Social	Serving a large population	18	12	31	0.22
	Serving a specific population within the hospital	18	8	15	0.58
	Improving the serves to the patient	6	8	27	0.07
	Patients’ preference	41	46	62	0.36
Technological	Lack of effective alternative technology	71	62	77	0.48
	Completion of a technology already exists at the hospital	18	27	23	0.78
	Innovation and Improving medical standards	18	31	46	0.14
	Improving hospital reputation	24	31	35	0.74
Economical	Efficiency	35	54	58	0.33
	Manpower savings	17	42	62	0.02
	Budget savings	24	46	24	14
	Technology that requires a significant investment in infrastructure	29	31	8	0.89
	Maintenance costs	12	15	4	0.37
	Extent education	29	12	23	0.33

No trend or statistically significant difference were noted in all the other criteria.

### Participant standpoints regarding the HTA process

#### Frequency of committee meetings

A total of 54% of the participants agreed that the committee should have discussions ad hoc, with 45% of the participants recommending a quarterly discussion. Only 1% thought the activity should be on an annual basis.

#### The relative importance of different players as committee members

Physicians were ranked as HI by 93% of the survey responders, nurses by 61%, bio-engineers by 69%, economists by 52%, patient representatives by 17% and information specialists by 9%.

#### Scope of committee deliberation

The existence of a separate HTA for each hospital was preferred by 84% of the participants, while 13% favoured a national process and 3% suggested a regional or grouped process.

## Discussion

Technology adoption in a hospital setting is usually based on considerations that include cost effectiveness analysis, feasibility, expedience, potential success and profitability [24]. Different countries have embraced a range of principles and involved stakeholders in order to accomplish an effective comprehensive process of HTA [25–27]. The current study analysed the viewpoints of technology-oriented hospital staff members toward the main aspects integrated in HTA. The data revealed that the relative weight varies among different hospital staff, according to their personal characteristics and professional standpoints. An amalgam of vectors, such as scientific, educational, cultural and managerial, merge to play a role in prioritizing the adoption of new technologies in a world of scarce resources [28].

Our study revealed, in accordance with the literature, that clinical aspects were considered as HI by most of the participants from all the profession groups, especially the criterion ‘lifesaving’ [29]. Economic aspects of HTA were ranked as HI only by half of the participants in our survey, while social and technological aspects were ranked as HI only by a relatively low percentage of the participants. Indeed, integrating social aspects into the HTA models is still a challenge.

The parameter ‘lack of effective alternative technology’ was also ranked as HI by a high percentage of the participants, independent of their profession. Nurses added the parameters ‘improving quality of life’, ‘improving patient function’, ‘increasing teamwork efficiency’ and ‘improving medical standards’. This was contrary to allied health professionals, engineers and administrators who were focused on the technological and economic aspects. In the literature, leading personal and professional values identified by nurses were human dignity, equality, prevention of suffering, responsibility towards patients and honesty [30]. This could explain the tendency of the nurses to give preference to patient needs. Other health professionals participated in our study and ranked their perspective on the importance of parameters; allied health professionals focused on lack of effective alternatives, engineers emphasised considering the need for significant investment in infrastructure, while administrators ranked patient experience as HI.

Physicians refer to availability and reasonableness of clinical care, while also taking into consideration the outcome of the technology used [31] when evalulating 'clinical effectiveness'. Other values, such as equity, justice, accountability or transparency, are rarely investigated as part of the decision-making process during HTA [32, 33], but rather observed on a global panorama. A study aimed to describe the role of social values in a priority-setting related to HTA processes and decision-making conducted in Australia revealed that, even when social values related to justice/equity were considered, no quantification of criteria weights for equity relative to other aspects has been published [34].

Interestingly, the age of the responders influenced their standpoints – the social aspect was ranked as HI by participants over 40 years of age. This may reflect a more advanced insight of the healthcare system as a whole [35]. Increasing effectiveness was ranked as HI by senior participants, as part of their accountability for team work. A significantly higher percentage of junior participants than senior participants ranked safety as HI, maybe due to a lack of experience or being intensely exposed to new standards of ‘patient safety’ programmes and hospital accreditation surveys [36].

Other ‘relative influence’ factors should be investigated while considering the adoption of a new technology, including scientific evidence and relevant available publications, pressure from senior physicians (being ‘early adopters’), pressure from the industry, resentment of the local professional teams, the need for additional investment, regulatory approval and type of provision (private/public mix) and profitability.

The results of our study demonstrated that the clinical aspect was favoured, especially among the medical staff. Similarly, the need for effective technologies alongside a lack of alternatives reflects demand. The viewpoint that the HTA mechanism should be instant, ad hoc or quarterly, demonstrates the participants’ perception of growing willingness to seek innovations. The relatively low ranking of economic parameters can be explained by the fact that the Israeli medical teams in hospitals are less exposed to financial considerations, as most of the medical care is covered by the national insurance law, as opposed to HTA in other countries [37].

The majority of the survey participants preferred HTA to be conducted by a multidisciplinary committee that is convened as needed. Most of the participants stated that physicians make the greatest contribution to the committee. In our opinion, this indicates the current perception that hospital technology implementation is conducted under autonomous accountability of the professional leadership [38].

The participants of the present study preferred that mostly physicians, and by a lesser rate additional medical professional, should be the members of the HTA committee. Interestingly, patient preferences were not at the core of consideration among all participant sectors. This may by secondary to a strong perception of paternalism and slow diffusion of ideas, such as the opportunity to amplify patient involvement, individual choice, shared decision-making and ‘patient experience’. Evaluating the awareness of ophthalmologists towards patient expectations revealed that only 17% stated that they are aware of patient expectations. The awareness was higher (45.5%) among ophthalmologists working primarily in a private clinic, those with a management position (30.1%) and those with more years of clinical experience (32%) [39]. Physicians in the private sector in the United Kingdom were found to be more experienced, both individually and collectively, in paying attention to patient culture and values [40]. In the United States of America, Medicare Hospitals that created collaborative cultures and higher physician engagement, accomplished a better technology implementation process and had a higher value-based purchasing initiative [41]. Thus, patient values and preferences cannot be ignored any longer and should be integrated into technology assessment.

Most of the responders in our study preferred that the mechanism of decision-making be conducted in each hospital separately (and not on a national or regional level). This is in agreement with additional studies demonstrating that HTA guidelines should be applied at a hospital level. According to Lettieri et al. [42], a technology can create a value for the hospital, in both the short- (mainly cost effectiveness) and long-term (development and accumulation of knowledge). In addition, technology adoption at the hospital level increases the rationality and the accountability of the technology assessment according to the hospital budget.

It has already been well established that comprehensive HTA is a necessity in evaluating and prioritising new healthcare technologies. The Office of Health Technology Assessment defined that the analysis should include the impacts of a particular technology on the individual and society regarding safety, efficacy, effectiveness and cost-effectiveness, as well as social, economic and ethical criteria [43]. Shemer and Siebzehner [44] have suggested a model that included evidence-based clinical, epidemiological and economic aspects, as well as financial resources and social, political ethical and legal considerations in the HTA. Cromwell et al. [45] found that healthcare decisions are based on criteria related to population health and organisational needs. Ethical issues, such as equity and accessibility, were also identified as important. In Israel, since 1999, HTA is conducted as part of expanding the National List of Health Services by a public National Advisory Committee that includes physicians, senior officials of the Ministry of Health and the Ministry of Finance, Sick-Fund directors and public representatives [46]. This committee bases its recommendations on similar clinical, economic and social parameters. Golan et al. [7] allocated three groups of criteria, representing a pluralistic approach, as need, appropriateness and clinical benefits, efficiency, and equality, solidarity and other ethical or social values. The current trend incorporates all these aspects into a multi-perspective multidisciplinary approach.

Using a multi-criteria decision analysis (MCDA) model developed to assist stakeholders’ decisions may be beneficial as the HTA mechanism becomes more complex. To increase effectiveness, the criteria should be sorted by numeric importance and performance weights to be combined into an overall score, which is used to rank the alternative treatments [47].

Not only is it necessary to identify the most effective criteria for HTA, there is also a worldwide need for formalization of health priority processes at both the national and local levels. Mirelman et al. [48] have compared decision-makers’ preferences at the country level according to a MCDA setting. The data revealed that MCDA is possible across a variety of countries. However, there are some voices opposing the worldwide formalisation of MCDA, arguing that the wide range of possible healthcare applications requires the use of specific MCDA for local, national and international levels [49].

Analysing the mechanism and methodology of the HTA process, the participants in the current study ranked the discussion in the committee as important, as they experienced the complexity in decision-making. This may be due to our methodology using ‘short format’/‘mini HTA’, but also due to the lack of solid published evidence in the rapidly changing world of medical technologies. Real world experience may assist in solving this barrier [50].

## Conclusions

Clinical, social, technological and economic considerations influence the decision-making process of technology adoption in a hospital arena. Various parameters, values, criteria and circumstances affected the process differently, according to the participants’ personal and professional characteristics. HTA should be carried out by utilising a MCDA, conducted by a multidisciplinary committee to balance and merge different forces. A nation-wide perspective in HB-HTA should be further discussed for beneficial achievements.

### Challenges and limitations

Compliance to policy-targeted surveys among hospital workers, especially among physicians, is relatively low (~20%) [51]. Thus, gathering the standpoints of leading players was a challenge. Using a friendly electronic questionnaire and conducting multiple (4–5) reminders resulted in a response rate of 89%.

The principal limitation of this survey is that it was conducted in one hospital, which has a long-term, well established mechanism of technology assessment. Thus, further studies in additional hospitals with different features should be performed.

Bioengineers and administrators are strongly involved in our HTA process, and were included in the survey. However, we found no literature reflecting the standpoints and values of this subgroup in adopting decisions.

The strength of this study is mainly the classification of multi-criteria aspects, as well as the multidisciplinarity of participants, resulting in a broad-minded point of view, thus suggesting that the MCDA approach could serve as a basis for establishing guidelines for the HTA process at a hospital as well as on a national level. Such models involve decision-makers explicitly weighting the criteria for the decision regarding the problem being addressed and rating the alternatives on the criteria, representing the relative importance of the criteria to the decision-maker. These weighted scores are then summed to produce the alternative’s total score with the ability to compare alternatives by ranking them relative to one another [52].

Indeed, the issue of developing unified methods to be used in the HTA process has been long discussed. In September 2006, the International Information Network on New and Emerging Health Technologies agreed that they would develop a methods toolkit to summarise the various approaches used in identification, filtration, prioritisation and subsequent assessment of new health technologies [53]. In 2011, the Agency for Healthcare Research and Quality Healthcare Horizon Scanning System published a Horizon Scanning Protocol and Operation Manual of a basic protocol and decision processes to be followed in order to identify new interventions that could have the greatest potential impact in each priority area [54].

## Data Availability

The datasets used and/or analysed during the current study are available from the corresponding author on reasonable request.

## Abbreviations

HB HTA: hospital-based health technology assessment
HI: highly important
HTA: health technology assessment
MCDA: multi-criteria decision analysis
